# Integrating network pharmacology and experimental verification to explore the pharmacological mechanisms of asparagus against polycystic ovary syndrome

**DOI:** 10.1186/s13048-023-01210-5

**Published:** 2023-07-01

**Authors:** Jinshan Xing, Xin Luo, Keran Jia, Shuang Liu, Shaokun Chen, Gan Qiao, Chunxiang Zhang, Jingyan Yi

**Affiliations:** 1 Department of Neurosurgery, The Affiliated Traditional Chinese Medicine Hospital of Southwest Medical University, Luzhou, 646000 Sichuan China; 2 Department of Pharmacology, School of Pharmacy, Nucleic Acid Medicine of Luzhou Key Laboratory, Southwest Medical University, Luzhou, 646000 Sichuan China; 3 Nucleic Acid Medicine of Luzhou Key Laboratory, Key Laboratory of Medical Electrophysiology, Ministry of Education & Medical Electrophysiological Key Laboratory of Sichuan Province, (Collaborative Innovation Center for Prevention of Cardiovascular Diseases), Institute of Cardiovascular Research, Southwest Medical University, Luzhou, 646000 Sichuan China; 4 Department of Reproductive Medicine, The Affiliated Hospital of Southwest Medical University, Luzhou, 646000 Sichuan China; 5 Department of Morphological Laboratory, School of Basic Medical Sciences, Southwest Medical University, Luzhou, 646000 Sichuan China; 6 Department of Medical Cell Biology and Genetics, School of Basic Medical Sciences, Nucleic Acid Medicine of Luzhou Key Laboratory, Key Laboratory of Medical Electrophysiology, Ministry of Education & Medical Electrophysiological Key Laboratory of Sichuan Province, Collaborative Innovation Center for Prevention of Cardiovascular Diseases), Institute of Cardiovascular Research, Southwest Medical University, Luzhou, 646000 Sichuan China

**Keywords:** PCOS, ASP, Network pharmacology, PRKCA

## Abstract

**Background:**

Polycystic ovary syndrome (PCOS) is a common reproductive endocrine disorder in women of reproductive age that still lacks effective treatment. Inflammation is one of the important features of PCOS. Asparagus (ASP) has anti-inflammatory, antioxidant, and anti-aging pharmacological effects, and its anti-tumor effects have been demonstrated in a variety of tumors. However, the role and mechanism of ASP in PCOS remain unclear.

**Methods:**

The active components of ASP and the key therapeutic targets for PCOS were obtained by network pharmacology. Molecular docking was used to simulate the binding of PRKCA to the active components of ASP. The effects of ASP on inflammatory and oxidative stress pathways in PCOS, and the regulation of PRKCA were examined by KGN, a human derived granulosa cell line. PCOS mouse model validated the results of in vivo experiments.

**Results:**

Network pharmacology identified 9 major active ingredients of ASP with 73 therapeutic targets for PCOS. Kyoto Encyclopedia of Genes and Genomes (KEGG) enrichment yielded 101 PCOS-related signaling pathways. The hub gene PRKCA was obtained after taking the gene intersection of the top 4 pathways. Molecular docking showed the binding of PRKCA to the 7 active components in ASP. In vitro and in vivo experiments showed that ASP alleviated the course of PCOS through antioxidant, anti-inflammatory effects. ASP can partially restore the low expression of PRKCA in the PCOS models.

**Conclusion:**

The therapeutic effect of ASP on PCOS is mainly achieved by targeting PRKCA through the 7 active components of ASP. Mechanistically, ASP alleviated the course of PCOS through antioxidant, anti-inflammatory effects, and PRKCA was its potential target.

## Introduction

Polycystic ovary syndrome (PCOS) is an inflammatory disease characterized by hyperandrogenemia and hyperinsulinemia [7]. Due to the lack of understanding of the origin and pathological mechanisms of PCOS, there is no effective treatment available [11, 25].

In recent years, Chinese medicine has adopted a multi-target and multi-path intervention strategy to play an overall regulatory and synergistic role, which has certain advantages for the prevention and individualized treatment of PCOS [13]. Clinical studies have shown that patients with PCOS are generally Clinical studies have shown that PCOS patients are generally in a state of chronic low-grade inflammation, and the inflammatory response can further promote the development of PCOS [17]. Asparagus (ASP) is a tuberous vegetable of the lily family whose roots and shoots are rich in many biologically active phytochemicals, including oligosaccharides, steroidal saponins, amino acid derivatives, and essential minerals [17]. ASP has been used to treat a variety of chronic inflammatory diseases and cancers. Polysaccharides, steroidal saponins and flavonoids extracted from ASP have been reported to be the main bioactive components with possible antitumor properties, acting mainly through apoptotic pathways and antimetastatic activity [3, 8, 15, 18, 21, 26]. Although ASP has been richly studied in tumors, its role in inflammatory diseases has not been elucidated.

**Fig. 1:**
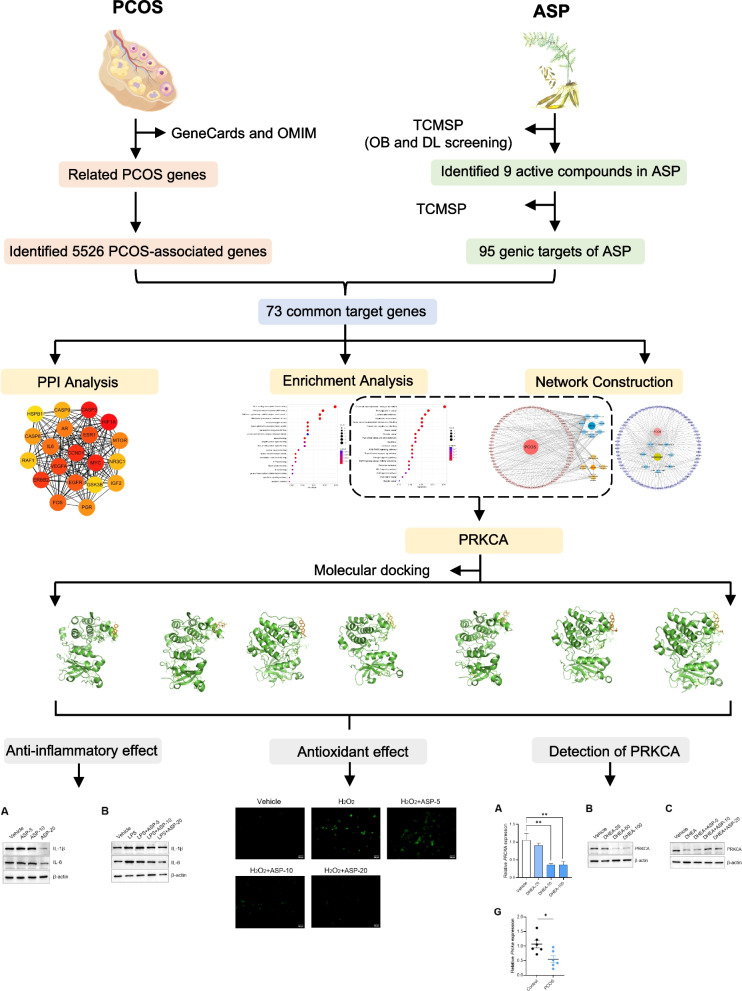
Schematic representation of the proposed mechanism in ASP against PCOS. Network pharmacology was applied to analyze the crucial components and key targets of ASP for the treatment of PCOS. Molecular docking revealed that 7 components had strong binding ability to the hub gene PRKCA. Cellular assays confirmed that ASP could alleviate the course of PCOS by inhibiting the caspase cascade pathway in GCs

As a new discipline based on systems biology, bioinformatics, and high-throughput histology, network pharmacology has achieved remarkable results in exploring the therapeutic mechanisms, screening of active ingredients and therapeutic targets of traditional Chinese medicine [14, 26]. In this study, we used network pharmacology combining molecular docking to discover the core target protein kinase C alpha (PRKCA), the core target of ASP for PCOS. PRKCA is a serine / threonine protein kinase and a member of the PKC family [9, 12]. We experimentally verified the mechanism by which ASP regulated oxidative stress and inflammatory response in granulosa cells (GCs) by targeting PRKCA. The flow chart of the study is shown in Fig. 1.

## Materials and methods

### Network pharmacology

Active ASP compounds were screened using the systematic Pharmacology Database of Traditional Chinese Medicine (TCMSP, http://tcmspw.com/tcmsp.php) [28]. Oral bioavailability and drug similarity were set to ≥ 30% and ≥ 0.18 using the pharmacokinetic information retrieval filter based on the TCMSP platform to obtain qualified herbal compounds. Chemical structures of the corresponding compounds were downloaded using the PubChem database (https://pubchem.ncbi.nlm.nih.gov/). The GeneCards database (https://www.genecards.org/) and the OMIM database (http://www.omim.org/ updated in 2022) were used to predict and screen PCOS targets. VennDiagram package in R was used to screen for common targets between ASP and PCOS-related targets.

Cytoscape V 3.7.0 (http://www.cytoscape.org/) software was used to construct drug compound-disease-target and drug compound-disease-pathway-target networks [20]. The STRING database (https://cn.string-db.org/) was used to construct protein–protein interaction (PPI) networks for common ASP and PCOS-related targets [22]. We selected high-confidence target protein interaction data with a score > 0.7. Top 20 PPI network proteins visualized with Cytoscape V 3.7.0 software. ClusterProfiler package in R was used for gene ontology (GO) analysis and Kyoto Encyclopedia of Genes and Genomes (KEGG) pathway analysis. The intersection selection of differential genes between crucial pathways was produced using the online tool (http://bioinformatics.psb.ugent.be/webtools/Venn/).

### Molecular docking

Molecular docking simulations were utilized to validate the binding of targets and corresponding compounds. Data on the construction of macromolecular protein target receptors were obtained through the RCSB PDB database (http://www.rcsb.org/) [1], and small molecule compounds were retrieved through the PubChem database and TCMSP. The macromolecular proteins downloaded from the PDB were excluded by water and ligands using PyMOL 3.7 software [19]. AutoDockTool 1.5.7 software was used to simulate the molecular docking of target proteins and corresponding compounds [23]. The genetic algorithm of the search parameters was used. PyMOL 3.7 software was used for network visualization.

### Cell cultures and treatment

KGN cells, a steroidogenic human granulosa cell-like tumor cell line was purchased from iCell Bioscience Inc (China) and identified by short tandem repeat (STR) profiling. Cells were maintained in Dulbecco’s modified Eagle medium F-12 supplemented with 10% fetal bovine serum and penicillin/streptomycin (100 units/ml) at 37 °C and 5% CO2. Cells were stimulated with DHEA (Macklin, China) for 48 h to simulate PCOS model in vitro. Vehicle group was treated with DMSO as control [27].

### Cell viability assay

ASP extract (10:1) was diluted in purified water to 2 g/ml. It was then filtered through a 0.22 μm filter and dispensed into sterile centrifuge tubes. The drug was stored at 4 °C. Cell viability was assayed using the cell counting kit-8 (CCK8) assay according to the manufacturer's protocol (Dojindo Molecular Technologies, Japan).

### Construction of PCOS models on mice

23-day-old C57BL/6 female mice were purchased from Beijing Vital River Laboratory Animal Technology Co., Ltd. (China). All animal procedures were conducted under the approval of the Animal Care and Use Committee of Southwest Medical University. C57BL/6 mice were housed in the SPF animal facility with controlled environment (22–24 °C, 60–70% relative humidity), a light / dark cycle (12 h light / 12 h dark), and free access to food and water. The PCOS mouse model was established by daily subcutaneous injection of 60 mg/kg DHEA (Macklin, China) for 23 days. The vehicle control group was injected with 0.09 ml aroma oil and 0.01 ml 95% ethanol daily. N = 6 mice each for vehicle and model groups. Microscopic analysis of the main cell types obtained from vaginal smears was used to determine the stage of the estrus cycle. On the last day of injection, the mice were executed by cervical dislocation and ovarian tissues were collected and set aside.

### Western blot analysis

Fresh cells were lysed with RIPA lysis buffer. Protein was separated by SDS-PAGE and transferred to PVDF membranes. Antibodies against PRKCA (D121138,1:1000), IL-1β (12703S,1:1000), IL-6 (21,865–1-AP, 1:1000) and β-actin (66,009–1-Ig,1:10,000) were used as the primary antibodies. HRP-conjugated antibodies against mouse or rabbit (1:10,000, Proteintech Group, Inc.) were used as the secondary antibodies. Immunoblot imaging was performed using the BIO-RAD ChemiDoc™ XRS + Molecular Imager. The western blot was normalized to β-actin.

### RNA extraction and RT-qPCR analysis

Total RNA was isolated by TRIzol reagent (Invitrogen). RNA was synthesized into cDNA using the HiScript II 1st Strand cDNA Synthesis Kit (Vazyme). Taq Pro Universal SYBR qPCR Master Mix (Vazyme) was used to conduct the qPCR analysis. QuantStudio™ Design & Analysis Software was used to analyze the samples. Gene expression was normalized to ACTB. The following primers were used:

- ACTB-Homo.
- 5′-CATGTACGTTGCTATCCAGGC-3′(Forward).
- 5′-CTCCTTAATGTCACGCACGAT-3′(Reverse).
- ACTB-Mouse.
- 5′-GGCTGTATTCCCCTCCATCG-3′(Forward).
- 5′-CCAGTTGGTAACAATGCCATGT-3′(Reverse).
- PRKCA-Homo.
  5′- ATGTCACAGTACGAGATGCAAAA-3′(Forward).
  5′- GCTTTCATTCTTGGGATCAGGAA-3′(Reverse).
  PRKCA -Mouse.
  5′- GTTTACCCGGCCAACGACT-3′(Forward).
  5′- GGGCGATGAATTTGTGGTCTT-3′(Reverse).

### The detection of reactive oxygen species (ROS) for GCs

Intracellular ROS was assayed using the Reactive Oxygen Species Assay Kit according to the manufacturer's protocol (Biosharp, China). Briefly, cells treated with 10 μM DCFH-DA, incubated in the dark for 1 h, then the cells were washed with pre cooled PBS and tested positive for DCFH-DA using fluorescence microscopy (Olympus, Japan)..

### Statistical analyses

All data are expressed as mean ± SEM. Statistical analysis was performed using GraphPad Prism 8 software. Differences between two independent groups were calculated using Unpaired Student's t-test and one way ANOVA with Tukey's multiple-comparisons test. *P* values less than 0.05 were considered statistically significant and are denoted as follows: * < 0.05, ** < 0.01, and *** < 0.001.

## Results

### Screening of the candidate genes of the active compounds in ASP

Using the TCMSP database, with oral bioavailability (OB) ≥ 30% and drug-like (DL) ≥ 0.18 as screening conditions, we obtained 9 components in ASP: methylprotodioscin_qt, Asparaside A_qt, 7-Methoxy-2-methyl isoflavone, beta-sitosterol, diosgenin, pseudoproto.dioscin_qt, quercetin and sitosterol (Table 1). We screened 95 drug target genes from 7 components (methylprotodioscin_qt and Asparaside A_qt had no target genes).

**Table 1:** ASP active components list

Molecule name	Mol ID	Oral Bioavailability (OB) %	Drug-like (DL)
Beta-sitosterol	MOL000358	36.91	0.75
Sitosterol	MOL000359	36.91	0.75
Methylprotodioscin_qt	MOL003889	35.12	0.86
Pseudoprotodioscin_qt	MOL003891	37.93	0.87
7-Methoxy-2-methyl isoflavone	MOL003896	42.56	0.20
Asparaside A_qt	MOL003901	30.60	0.86
Stigmasterol	MOL000449	43.83	0.76
Diosgenin	MOL000546	80.88	0.81
Quercetin	MOL000098	46.43	0.28

### Screening of candidate targets for PCOS

**Fig. 2:**
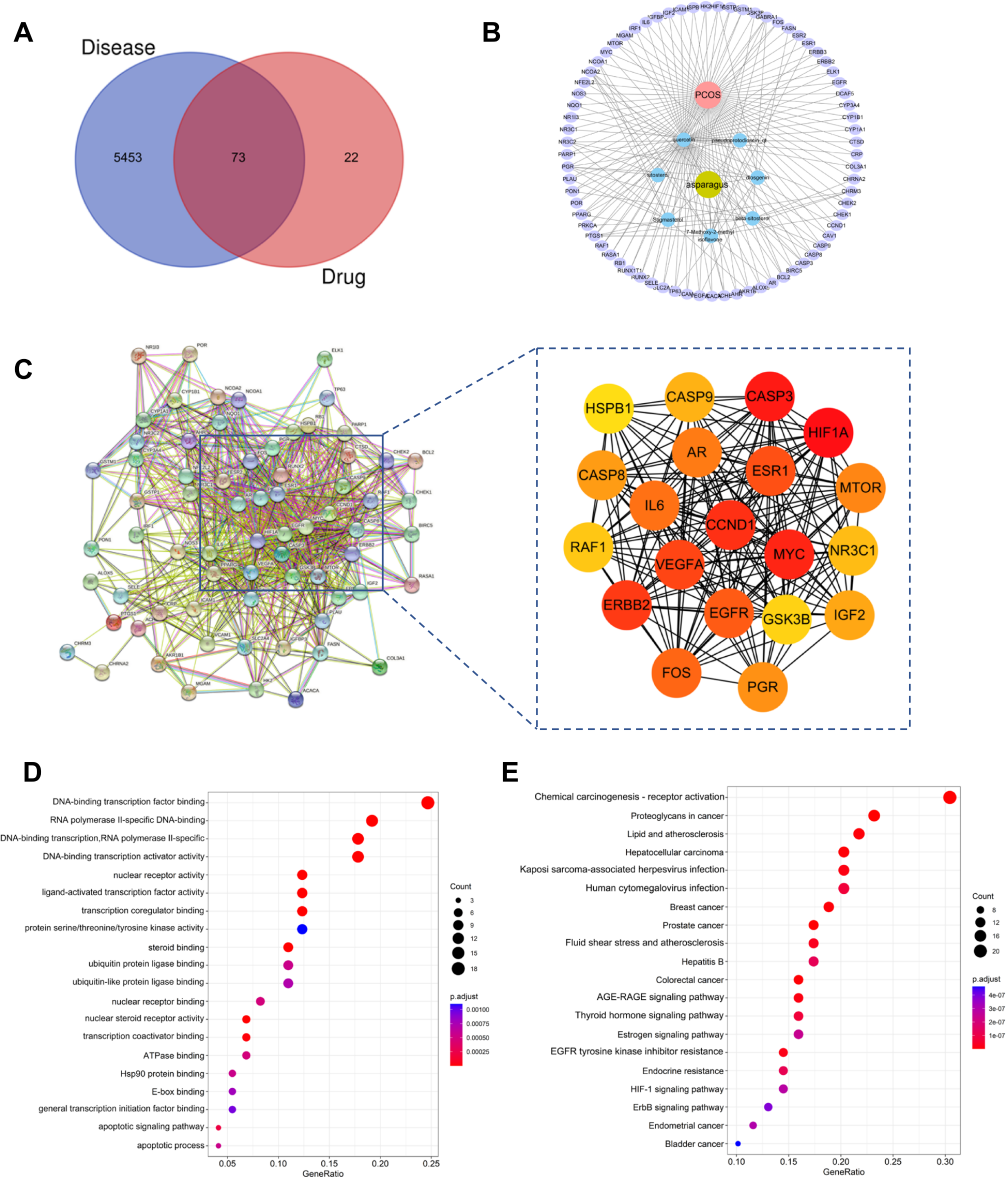
Network pharmacological analysis of ASP for PCOS. **A** Disease-drug Venn diagram. **B** Composition-target-disease network of ASP. The red circle represents the disease; yellow circle is for traditional Chinese medicine; Blue circles represent the 7 monomers; violet circles represent targets. **C** The 20 hub genes identified from the PPI network. The darker the color is, the more significant the gene. GO function enrichment (**D**) and KEGG enrichment (**E**) bubble diagram result of ASP in the treatment of PCOS. The color scale indicates the different thresholds of p values and the size of the dots represents the number of genes corresponding to each term

To mine targets associated with PCOS, we obtained 4982 and 328 disease-related genes from Genecards and OMIM databases, respectively, using PCOS as an index keyword. After removing duplicates and combining the search results, a total set of 5526 PCOS-associated genes was obtained. We used Venn diagrams to intersect ASP key component target genes and disease-associated genes to obtain 73 ASP-PCOS common targets (Fig. 2A).

### Network pharmacology analysis of ASP in PCOS

We imported 7 active ingredients and 73 drug-disease key target genes into Cytoscape 3.7.0 software to construct a visualized compounds-disease-target network (Fig. 2B). It can be seen that the active ingredient with the most target genes in ASP is quercetin. In order to investigate the relationship between common targets, we imported 73 genes into STRING online database to construct a PPI network (Fig. 2C). Then the network files were imported into Cytoscape 3.7.0 software for visualization. The top 20 key genes were screened using the MCC algorithm of cytohubba plugin, which included 3 important apoptotic pathway genes, CASP3, CASP8 and CASP9 (Fig. 2C). To further investigate the biological functions of the above candidate targets, we performed GO and KEGG enrichment analysis by R software. The top ranking among the 79 GO analyses was DNA-binding transcription factor binding, indicating that ASP may play an important function in the nucleus (Fig. 2D). Having analyzed 101 KEGG pathways, we speculate that ASP may act through Chemical carcinogenesis—receptor activation (Fig. 2E).

### Screening of hub genes for ASP targeting PCOS

**Fig. 3:**
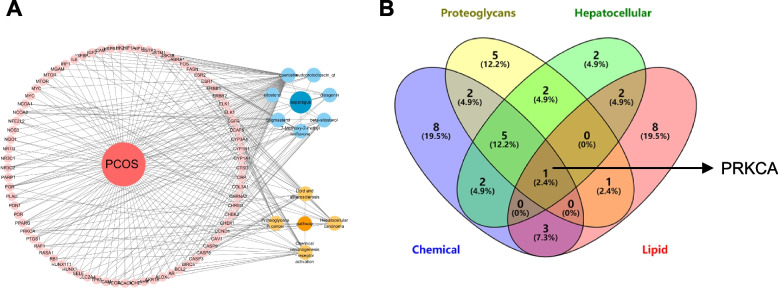
Screening for hub gene PRKCA. **A** Composition-target-disease-pathway network of ASP. The dark red circle represents the disease; dark blue circle is for traditional Chinese medicine; blue circles represent compound component; red circles represent targets; yellow circles represent core pathways. **B** Core pathways Venn diagram. The arrow indicates the hub gene common to the 4 pathways

KEGG pathways, active ingredients and 73 targets were imported into Cytoscape 3.7.0 software to construct a visualized compounds-disease-target-pathway network (Fig. 3A). The top 4 KEGG pathways filtered according to p-value and count numbers were Chemical carcinogenesis—receptor activation, Proteoglycans in cancer, Lipid and atherosclerosis and Hepatocellular carcinoma (Table 2). The junction gene PRKCA was obtained by intersecting a group of genes in the above pathways (Fig. 3B).

**Table 2:** Top 4 KEGG pathways

Description	*P* value	Q value	Count
Chemical carcinogenesis receptor activation	1.71E-17	2.02E-15	21
Proteoglycans in cancer	8.34E-12	4.92E-10	16
Hepatocellular carcinoma	8.29E-11	2.43E-09	14
Lipid and atherosclerosis	2.03E-10	3.43E-09	15

### Molecular docking simulates the binding of PRKCA with ASP active ingredients

**Fig. 4:**
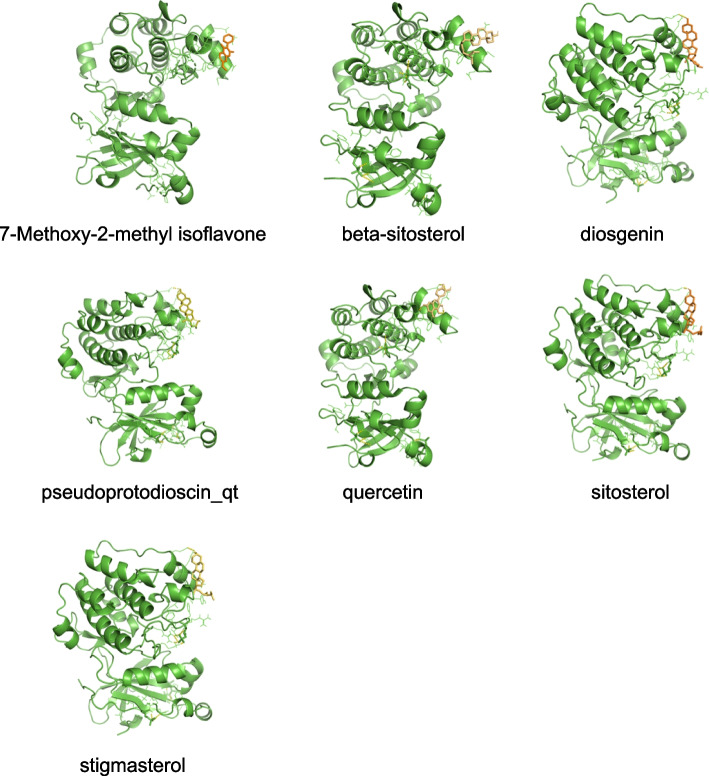
Molecular docking models of PRKCA binding to the 7 active components of ASP. PRKCA binds to 7-Methoxy-2-methyl isoflavone, beta-sitosterol, diosgenin, pseudoprotodioscin_qt, quercetin, sitosterol and stigmasterol

To explore the association of PRKCA with ASP. We performed molecular docking simulations of PRKCA with 7 active ingredients of ASP. The binding fractions of all components were less than -4 kcal/mol, suggesting a good binding capacity (Table 3). The binding schematic is shown in Fig. 4.

**Table 3:** Molecular docking score

Molecule name	Docking score (kcal/mol)	Protein
Beta-sitosterol	-5.21	PRKCA
Sitosterol	-5.47	PRKCA
Pseudoprotodioscin_qt	-5.91	PRKCA
7-Methoxy-2-methyl isoflavone	-4.96	PRKCA
Stigmasterol	-6.28	PRKCA
Diosgenin	-6.15	PRKCA
Quercetin	-4.77	PRKCA

### ASP ameliorates inflammatory response and oxidative stress in KGN cells.

**Fig. 5:**
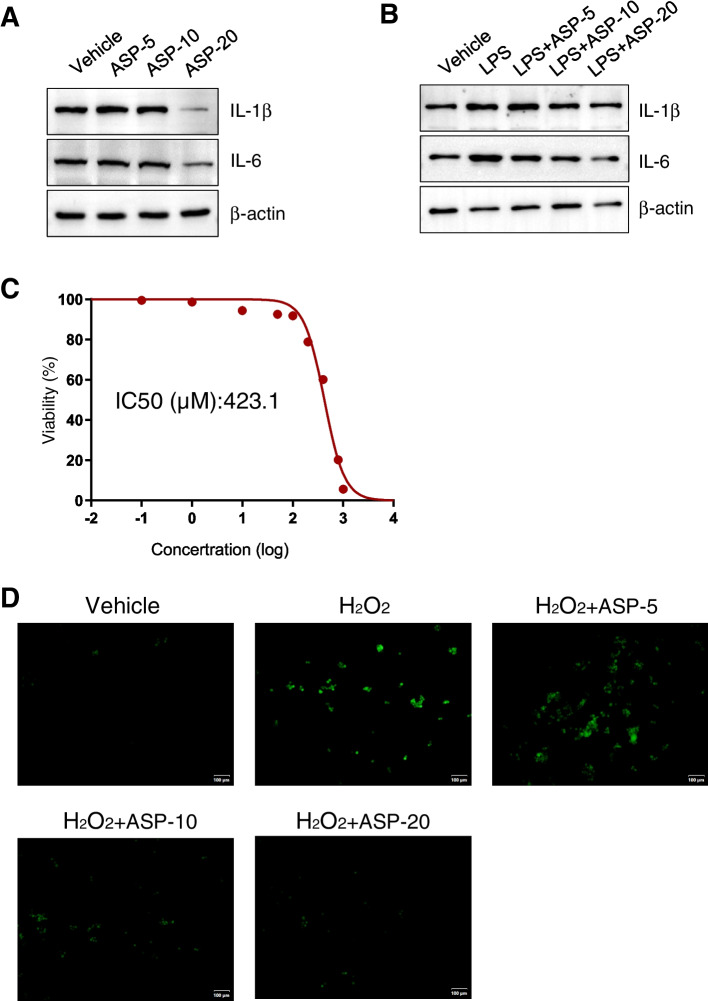
ASP ameliorates inflammatory response and oxidative stress in KGN cells. **A** Western blot was performed to detect the expression of IL-1B and IL-6 in ASP-treated KGN cells. β-actin was used as a normalization standard. **B** The effects of ASP on inflammatory factors in LPS treated KGN cells were determined by Western blot analysis. β-actin was used as a normalization standard. **C** CCK-8 assay to examine the viability of KGN cells after treatment with different concentrations of H_2_O_2_, IC50 value was calculated. **D** DCFH-DA staining showed the accumulation of ROS. Scale bar, 100 μm

We treated KGN cells with ASP to evaluate its effect on inflammation and oxidative stress. Western blotting was performed using a solution containing ASP (5 mg/mL, 10 mg /mL and 20 mg/mL) of culture medium for KGN cells. After 24 h, proteins were extracted to detect the expression of inflammatory factors. We found that ASP significantly reduced the expression of anti-inflammatory cytokines IL-1B and IL-6 (Fig. 5A). Meanwhile, ASP inhibited LPS induced inflammatory responses in a dose-dependent manner (Fig. 5B). To explore the role of ASP on oxidative stress, we established a model of H_2_O_2_ induced oxidative stress in which KGN cells were treated with various concentrations of H_2_O_2_. CCK-8 experiments showed that cell viability decreased with increasing H_2_O_2_ concentration (IC50 value of 423.1 μM) (Fig. 5C). Based on the established model of oxidative stress injury, cells were treated with ASP (5 mg /mL, 10 mg /mL and 20 mg/mL) for 24 h to detect ROS levels in KGN cells. The results showed that the fluorescence intensity in the injury model group was significantly higher than that in the control group, and ASP dose dependently reduced ROS accumulation in KGN cells (Fig. 5D).

### ASP reverses DHEA induced decrease of PRKCA in PCOS granulosa cells

**Fig. 6:**
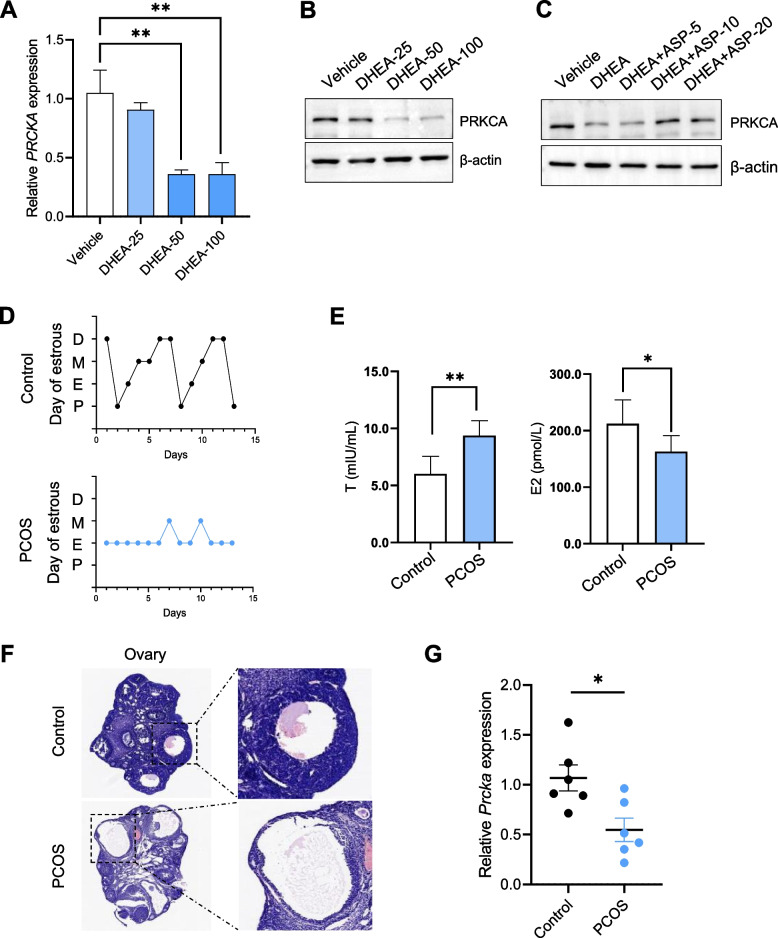
ASP reverses DHEA induced decrease of PRKCA in PCOS granulosa cells. **A**-**C** qRT-PCR and Western blot were performed to detect the expression of PRKCA in KGN cells. β-actin was used as a normalization standard. **D** Estrus status of mice in the DHEA and control groups (n = 6 per group). **E** ELISA assay for T and E2 in mouse serum. **F** Histological staining of ovaries from DHEA and control mice. **G** qRT-PCR was performed to detect the expression of PRKCA in PCOS mouse models

We stimulated KGN cells with DHEA to mimic PCOS. After DHEA treatment, PRKCA in KGN cells appeared significantly decreased, and this trend could be alleviated by ASP (Fig. 6A-C). It is suggested that ASP may play an important role in PCOS by targeting PRKCA. Next, we established a DHEA induced PCOS mouse model. We examined the characteristic indicators of PCOS. Mice in DHEA group exhibited obvious estrous cycle disturbance (Fig. 6D), increased serum T and decreased serum E2 (Fig. 6E). Further histological observation showed that the ovaries of mice in the DHEA model group appeared multiple cystic follicles (Fig. 6F). We then examined the expression of PRKCA in the control and PCOS groups. The results showed that PRKCA was significantly decreased in the PCOS group, consistent with the in vitro results (Fig. 6G).

## Discussion

PCOS is considered a chronic inflammatory disease that poses a serious threat to the health of women worldwide [6, 17]. Women with PCOS are usually characterized by obesity, hyperandrogenemia and hyperinsulinemia [7]. More and more herbal formulas are being widely used to treat a variety of diseases because of their multiple targets [13]. In this study, we combined network pharmacology and experimental validation to explore the potential molecular mechanisms of ASP for the treatment of PCOS.

Exogenous administration of androgens can induce the PCOS phenotype in experimental animals. The DHEA-induced model has similar reproductive characteristics to PCOS, such as loss of the motility cycle, elevated luteinizing hormone and testosterone levels, and a significant reduction in the number of mature follicles and corpus luteum, making it an ideal model for PCOS research [4, 10, 24]. Since GCs dysfunction is an important cause of follicular abnormalities in PCOS, we chose human ovarian granulosa cell tumor cells KGN to mimic PCOS in in vitro [5]. PRKCA is a serine / threonine protein kinase and a member of the PKC family [9, 12].

ASP is a tuberous vegetable of the lily family, with antibacterial and anti-inflammatory effects, and has significant therapeutic effects on bronchitis, pneumonia, tuberculosis, and emphysema caused by various coughs and phlegm, and asthma [3]. The anticancer activity of polysaccharides, saponins and flavonoid extracts from ASP has been reported in a variety of cancers, such as lung cancer, hepatoma and ovarian cancer [3, 8, 15, 21, 26]. Mechanistically, ASP exerted pro-oxidant activity and had a combined lethal effect with menaquinone in breast cancer [16]. Methanolic extracts of ASP could regulate apoptosis by activating the TRAIL death receptor pathway in colorectal cancer [2]. In other diseases, ASP inhibited TNFα induced apoptosis in Hep G2 cells to prevent alcohol-induced hepatotoxicity [3]. Meanwhile, we identified 3 important genes of apoptosis pathways, CASP3, CASP8 and CASP9, among the top 20 targets of PPI network with significant correlation. However, the flow cytometry results suggested that ASP did not significantly decrease the apoptosis of KGN cells stimulated by DHEA (Results not shown). One possible explanation is that PCOS patients are usually a long-term stimulus, whereas in vitro cell experiments are relatively short-term mimics and may not necessarily illustrate the role of ASP in apoptosis. Interestingly, we found that ASP significantly reduced LPS induced inflammatory response and H_2_O_2_ induced oxidative stress, indicating that ASP may play a therapeutic role in GCs of PCOS through an anti-inflammatory, antioxidant pathway. On the other hand, we found that PRKCA was significantly decreased in the PCOS group, and ASP could partially restore PRKCA expression. This may suggest a protective role of ASP on PCOS via regulation of PRKCA, which deserves our further exploration.

In this study, we discovered for the first time the therapeutic effect of ASP on PCOS through network pharmacology and experimental validation, and mined PRKCA, a potential target of ASP. This study provides new insights into the herbal treatment and target development for PCOS.

## Conclusion

In summary, we screened the hub gene PRKCA for ASP treatment of PCOS by network pharmacology and experimental validation. The mechanism by which ASP exerts protective effects in PCOS through an anti-inflammatory, antioxidant pathway was elucidated. The means of data mining employed in this study proved to be rapid and efficient. Our results can inform future similar studies.

## Data Availability

The data used to support the findings of this study are available from the corresponding author upon request.

## Abbreviations

PCOS: Polycystic ovary syndrome
ASP: Asparagus
GCs: Granulosa cells
GO: Gene ontology
KEGG: Kyoto Encyclopedia of Genes and Genomes
PPI: Protein–Protein Interaction
ROS: Reactive oxygen species
